# Long-Term Survival of Methotrexate as First-Line Therapy in Rheumatoid Arthritis, Psoriatic Arthritis and Undifferentiated Arthritis

**DOI:** 10.3390/jcm13247540

**Published:** 2024-12-11

**Authors:** Fabio Massimo Perrotta, Pasquale Ambrosino, Ennio Lubrano

**Affiliations:** 1 Academic Rheumatology Unit, Department of Medicine and Health Sciences “Vincenzo Tiberio”, University of Molise, 86100 Campobasso, Italy; 2 Istituti Clinici Scientifici Maugeri IRCCS, Scientific Directorate of Telese Terme Institute, 82037 Telese Terme, Italy; 3 Istituti Clinici Scientifici Maugeri IRCCS, Cardiac Rehabilitation Unit of Telese Terme Institute, 82037 Telese Terme, Italy

**Keywords:** rheumatoid arthritis, psoriatic arthritis, treatment, disability, exercise, rehabilitation, outcome, methotrexate

## Abstract

**Background/Objectives**: In the era of biotechnological drugs, methotrexate (MTX) still represents the first-line treatment in chronic inflammatory arthritis, such as rheumatoid arthritis (RA), psoriatic arthritis (PsA), and undifferentiated arthritis (UA). The aim of our study was to evaluate the persistence of MTX as a first-line treatment in a group of patients with chronic inflammatory arthritis. **Methods**: We conducted a retrospective analysis of a database of outpatients diagnosed with RA, PsA, or UA who visited our Rheumatology Clinic from January 2014 to January 2022. Key demographic and clinical data, including information on comorbidities and treatments, were routinely collected. Kaplan–Meier (KM) curves were used to determine the persistence of MTX during follow-up. **Results**: A total of 242 patients with chronic inflammatory arthritis who initiated MTX as first-line therapy and had available clinical data were included. Of these, 130 (53.7%) had RA, 82 (33%) had PsA, and 30 (16.3%) had UA. Overall, the survival rate of MTX at 24 months of follow-up was approximately 60%, while at 48 months and 96 months, it was 40% and 20%, respectively. A statistically significant difference was found between RA and PsA compared to UA (Chi-square test = 14.84; *p* = 0.001). When comparing the KM survival curves of MTX between male and female patients, obese and non-obese individuals, as well as older (age ≥ 50 years) and younger patients (age < 50 years), no statistically significant differences were observed in any of the comparisons. **Conclusions**: Our study confirmed the efficacy and overall safety of MTX in RA and PsA, with good persistence even over the long term.

## 1. Introduction

In the era of biologic (b) and targeted synthetic (ts) disease-modifying anti-rheumatic drugs (DMARDs), methotrexate (MTX), a conventional synthetic (cs) DMARDs, still represents the first-line treatment in inflammatory arthritis such as rheumatoid arthritis (RA) and psoriatic arthritis (PsA) [1,2]. Despite the huge number of studies demonstrating the efficacy of b/tsDMARDs in achieving clinical response, remission, or low disease activity in RA and PsA, MTX remains regarded as the “anchor” drug [3]. In particular, MTX is uniquely capable of significantly modifying disease outcomes, particularly in RA, where it has been shown to induce Disease Activity Score 28 (DAS28) remission in approximately 20–30% of early-stage patients [3]. Data on its efficacy in PsA are scarcer, but a recent randomized controlled trial showed that 22.9% of MTX-monotherapy patients achieved minimal disease activity after 24 weeks of treatment [4], with previous observational studies showing a remission rate of about 25% [5]. In fact, the European Alliance of Associations for Rheumatology (EULAR) recommendation for the treatment of RA still recommends the use of MTX (preferred over other DMARDs) as first-line treatment, and the Group for Research and Assessment of Psoriasis and Psoriatic Arthritis (GRAPPA) recommendation strongly endorses the use of MTX for active peripheral arthritis and conditionally recommends MTX for the treatment of enthesitis, dactylitis, and skin involvement [1,2]. MTX mechanism of action involved several cellular pathways, including inhibition of dihydrofolate reductase, reduction of monocytic and lymphocytic cytokine production, and reduction of specific cytokines via adenosine binding on A3 receptor and increased levels of cAMP. Historically, four well-designed, blinded, placebo-controlled studies published in 1984 and 1985 introduced the use of MTX in the treatment of RA, with other observational studies that confirmed its importance as a disease-modifying drug [6,7,8,9], while evidence of the efficacy in PsA and undifferentiated arthritis is still scarce and based mainly on observational studies and trials with low quality of evidence. Moreover, the MIPA study, although with some limitations, showed no better efficacy with respect to placebo in the achievement of clinical response in patients with PsA [10]. Despite its wide use, to date, fewer reports have been published regarding the long-term persistence of MTX in patients with inflammatory arthritis in daily clinical practice. Moreover, few studies have evaluated the clinical factors that may contribute to long-term persistence. Finally, the persistence of MTX may vary considerably across different studies [11,12,13,14,15,16,17,18,19,20].

Furthermore, we reviewed the literature (by searching on PubMed and Embase the terms methotrexate, persistence, survival rate, arthritis, rheumatoid arthritis, psoriatic arthritis, and undifferentiated arthritis) from the past three years and found only one retrospective study addressing MTX persistence in RA [12], while no recent studies have been published for PsA. In fact, in recent years, the research focus has mainly shifted toward the persistence of b/ts DMARDs, either in monotherapy or in combination with MTX. However, the survival of MTX, which is still considered the “anchor” drug in inflammatory arthritis, has not been addressed.

In this context, the objective of our study was to assess the persistence of MTX as first-line treatment in a group of patients with chronic inflammatory arthritis (RA, PsA, and undifferentiated arthritis) in the context of clinical practice. The secondary aim was to identify potential clinical factors associated with long-term persistence.

## 2. Methods

We retrospectively reviewed the clinical records of outpatients with chronic inflammatory arthritis who attended the Rheumatology Clinic at the Department of Medicine and Health Sciences “Vincenzo Tiberio” at the University of Molise, Italy, between January 2014 and January 2022. For the purpose of this study, inclusion criteria were at least one of the following: clinical diagnosis of RA (classified with the 2010 EULAR criteria) [21]; clinical diagnosis of PsA (classified with the CASPAR criteria) [22]; clinical diagnosis of undifferentiated arthritis (UA), namely patients with inflammatory joint disease that do not fit the criteria for a specific diagnosis. Patients were also included if they had at least 2 years of follow-up in our unit, starting treatment with oral, intramuscular, or subcutaneous first-line MTX during the considered period. The exclusion criteria included the following: age < 18 years; initiation of MTX in combination with other cs/b/ts DMARDs; and incomplete clinical data, including missing information on MTX persistence or MTX dose/route of administration.

This study adhered to the Strengthening the Reporting of Observational Studies in Epidemiology (STROBE) guidelines [23] where applicable and followed the Declaration of Helsinki set forth by the World Medical Association. The Institutional Review Board of the University of Molise, Campobasso, Italy, reviewed and approved this study protocol (No. 24/2017). The need for informed consent was waived, given the retrospective nature of this study.

### 2.1. Study Protocol

We designed a retrospective cohort study on patients with RA, PsA, or UA who began first-line treatment with MTX, evaluated at various time points throughout the observation period. Specifically, in line with current clinical practice for this patient group at our center, study participants were regularly examined every 3–6 months, and data were systematically collected and stored in an electronic database. To be included in this study, the minimum follow-up duration was required to be at least 2 years.

Persistence, defined as the duration of time that patients continued their MTX treatment without discontinuation, served as the primary outcome measure of our study, allowing us to evaluate the effectiveness of MTX as a first-line therapy for chronic inflammatory arthritis but also the clinical factors influencing patients’ adherence to treatment over time.

### 2.2. Study Procedures

Data collected for all patients in the Outpatient Clinic database included diagnosis, sex, age, smoking habit, body mass index (BMI), symptoms duration, disease duration, clinical measures of disease activity in inflammatory arthritis, C-reactive protein (CRP) levels (collected within 4 weeks from the patient’s visit), comorbidities, and current/past treatment. Obesity was defined by the presence of a BMI greater than 30 kg/m^2^. 

For clinical assessment of disease activity, the tender (out of 68) and swollen joint count (out of 66) were evaluated in all patients. Briefly, the tender joint count was used to assess pain in 68 joints when each joint is palpated and pressure is applied, indicating inflammation and sensitivity. Additionally, the swollen joint count measured the number of joints that were visibly swollen, reflecting active inflammation and fluid accumulation in 66 joints. Both tender and swollen joints were recorded numerically, with higher scores reflecting greater levels of active inflammation. Collectively, these measures were used in evaluating treatment response and guiding clinical management for patients with RA, PsA, and UA.

### 2.3. Statistical Analysis

Statistical analysis was performed using SPSS 26.0 (IBM, Chicago, IL, USA). Continuous variables were expressed as the median and interquartile range (IQR), and categorical variables were expressed as numbers and percentages. Kaplan–Meier (KM) survival curves were plotted to determine the persistence of MTX during the follow-up. In KM survival curve calculation, we entered the time until the subject was “censored” or the “event” (discontinuation of treatment) occurred. The difference between survival curves was determined by the log-rank test. To evaluate the clinical factors influencing persistence, we also stratified patients by sex (male vs. female), body weight (obese vs. non-obese), and age (≥50 years vs. <50 years). This allowed us to compare the KM survival curves according to the relevant clinical or demographic variables.

Cox regression was used to determine factors associated with time-to-event, the event being the discontinuation of MTX. Univariate Cox regression was performed to estimate the potential factors of MTX discontinuation. Variables included in the Cox regression were age, sex, smoking, BMI, dose, and duration of steroid treatment. *p*-values < 0.05 were considered significant. 

## 3. Results

**Figure 1:**
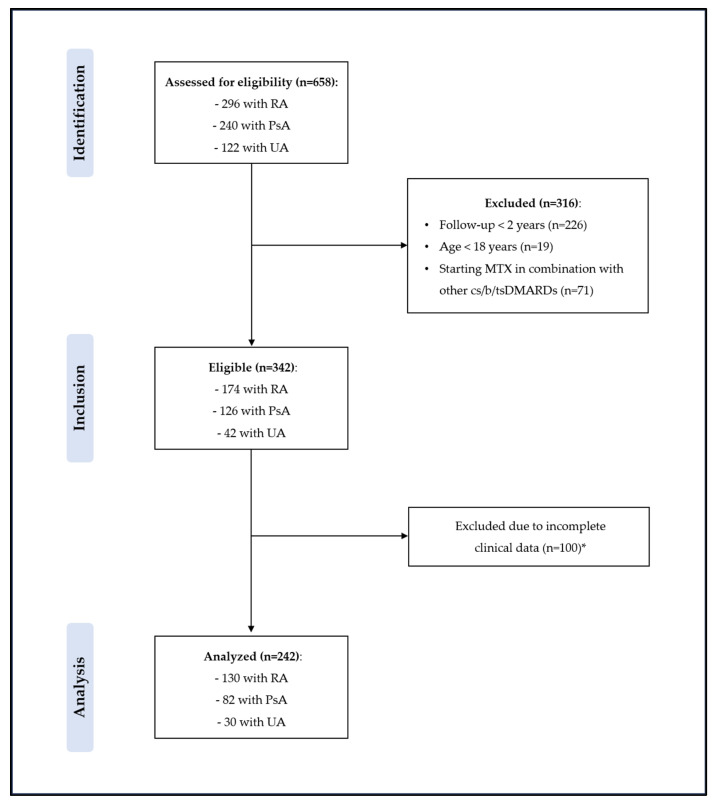
Flowchart of this study’s participants. * 100 participants excluded due to incomplete data, including height, weight, tender/swollen joint counts, and missing information about MTX persistence or MTX dose/route of administration. Abbreviations: MTX: methotrexate; RA: rheumatoid arthritis; PsA: psoriatic arthritis; UA: undifferentiated arthritis; cs/b/tsDMARDs: conventional synthetic, biologic and targeted synthetic disease-modifying anti-rheumatic drugs.

A total of 658 participants with inflammatory arthritis were screened for eligibility, of which 296 (45%) had RA, 240 (36%) had PsA, and 122 (19%) had UA. Of these, 316 (48%) were excluded for not meeting the enrollment criteria specified in our protocol, including 226 with a follow-up duration of less than 2 years, 19 under the age of 18, and 71 who started MTX in combination with other cs/b/tsDMARDs. Therefore, patients with chronic inflammatory arthritis who started MTX as first-line therapy eligible for inclusion in the analysis were 342. Of these, 100 (29.2%) were excluded due to incomplete data, including height, weight, tender/swollen joint counts, missing information about MTX persistence, or MTX dose/route of administration. Therefore, a total of 242 subjects with chronic inflammatory arthritis were considered in the final analysis (Figure 1), of which 130 (53.7%) had RA, 82 (33%) had PsA, and 30 (16.3%) had UA.

Table 1 shows the clinical characteristics of patients enrolled. As expected, in patients with PsA we found a higher prevalence of male sex, while median age was slightly lower compared to patients with RA. In patients with UA, the percentage of males between the two sexes was comparable. Median BMI was higher in patients with PsA, as confirmed by the higher proportion of obesity (20.2% in PsA vs. 12.9% in RA vs. 7.1% in UA). Treatment with MTX was started in all patients as a first-line drug (95% of patients started subcutaneous MTX, while 5% oral MTX). No patients started intramuscular MTX. The median starting dose of MTX was 15 mg per week (range: 10–20 mg). No statistically significant differences were found in MTX dose between the three groups. All patients starting MTX had active peripheral arthritis.

**Figure 2:**
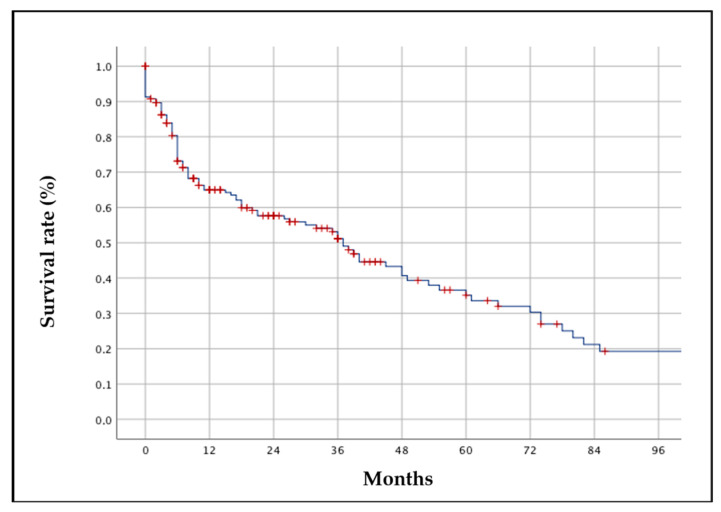
Overall methotrexate (MTX) survival rate at 96 months.

Figure 2 illustrates the persistence of methotrexate (MTX) in our patient group. Notably, the survival rate at 48 months was 40%, while it declined to 20% at 96 months. During the follow-up, of the 242 patients with MTX treatment, 52 patients suspended the drug due to intolerance or adverse events (mainly an increase in liver transaminases or gastrointestinal tolerability). The remaining 194 patients were suspended MTX due to primary and secondary inefficacy and switched to b/ts DMARDs monotherapy or disease remission. 

**Figure 3:**
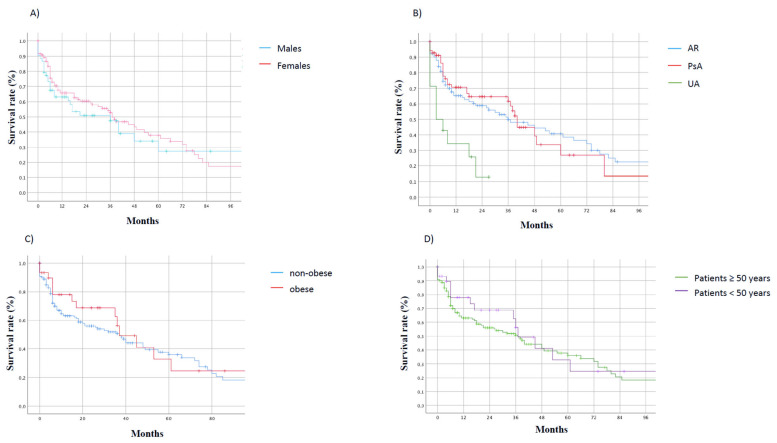
Kaplan–Meier (KM) curves. (**A**) Methotrexate (MTX) survival in patients with RA, PsA, and UA; (**B**) MTX survival in males and females; (**C**) MTX survival in obese vs. non-obese individuals; (**D**) MTX survival in patients ≥ 50 years and patients < 50 years.

Figure 3A compares the KM survival curve of MTX in patients with RA, PsA, and UA. A statistically significant difference was found between RA and PsA compared to UA (Chi-Square test = 14.84; *p* = 0.001). Figure 3B–D compares the KM survival curve of MTX in male vs. female sex, obese vs. non-obese individuals, and older vs. younger (age ≥ 50 vs. age < 50 years) patients. No statistically significant differences were found for all comparisons. At Cox regression, no predictors associated with MTX discontinuation were found (Table 2).

## 4. Discussion

The results of this study confirmed the validity of MTX treatment in chronic inflammatory arthritis, showing overall good persistence at 2-, 4-, and 8-year follow-up intervals and highlighting some differences across the groups examined. The lower survival rate of MTX in UA could be explained by several factors, including changes in diagnosis with other conditions not requiring the use of MTX or the presence of self-limiting inflammatory arthritis. In fact, of the 30 patients with UA, 14 had a diagnostic change (axial spondylarthritis, crystal arthritis, osteoarthritis, and fibromyalgia) during follow-up.

These findings contribute to a clinical and scientific landscape increasingly focused on targeted pharmacological therapies aimed at specific molecular targets or endotypes. While this trend is evident across many clinical fields—particularly in respiratory diseases—it is important to remember that MTX still represents the first line of treatment for chronic inflammatory arthritis, despite the recent rise and development of new biologic drugs. In fact, since its approval for use in arthritis in 1988, MTX has continued to be the drug of choice for RA, PsA, and other chronic inflammatory arthritis, often used in combination with biologic drugs to enhance their effect.

For what concerns the persistence of MTX therapy in patients with RA, we found a survival rate of 65% at 12 months, in keeping with that found by Lie E. et al. in 2009, where a persistence of 60% was demonstrated at 12 months [11]. On the other hand, in a recent retrospective study, the survival rate of MTX in RA at 5 years was 81%, which was quite higher compared to our study [12]. Overall, in the literature, the long-term survival rate of MTX in RA ranges from 46% (8) to 81% [11,12,13,14,15,16,17,18,19,20,24]. However, there are different factors to consider when dealing with MTX in RA that can justify the overall lower rate of persistence in our study with respect to the results by Dhir et al. [12]. First, with the availability of new b/tsDMARDs, which can be used in monotherapy, MTX withdrawal may become more frequent. Secondly, there are several differences in the methodology, patients’ population, and time to assessment among the different studies. Finally, the possibility of achieving remission and even drug-free remission may lead to a rapid withdrawal of csDMARDs. 

In fact, the persistence of MTX is generally higher in studies performed before the massive introduction of b/tsDMARDs in clinical practice, as reported in the Dutch cohort [16].

In our study, the persistence of MTX in patients with PsA was 70% at 12 months, which is slightly higher compared to the study by Lie E. et al., where the persistence was about 57% at 12 months [11], while at 36 months, the survival rate was 64%, similar to that found in the study by Ricci M. et al. (59.8% at 36 months) [20]. Moreover, the results of our study were in keeping with those reported in this Norway observational study, in which the rate of persistence in both RA and PsA seems to be similar and confirms that, although there is scarce evidence of the efficacy in PsA, the retention rates do not differ from RA [11]. This may confirm the effectiveness of the drug.

Overall, our study showed some interesting findings: treatment persistence dropped in the first 12 months of treatment, and this may be justified by the early onset of side effects or intolerance. After the first year, discontinuation of treatment could probably be associated with loss of efficacy or other reasons such as achievement of remission.

This finding is only partially in keeping with other experiences in the literature. In fact, in a retrospective study evaluating patients starting MTX as first-line therapy, MTX discontinuation occurs sooner in patients with PsA with respect to RA but with greater rates of both early discontinuations, as shown in our study in which the survival rates seem to drop in the first months [25].

Other important findings of our study were the overall similar rate of survival in both sexes, which could be justified by the high median age of our patients (post-menopausal women) and a similar rate of survival in older patients and obese individuals compared to younger and non-obese. These findings further support the effectiveness and safety of MTX in these peculiar populations and are in keeping with the study of Bologna et al. in which similar findings were found in patients older or younger than 65 years [24].

Our study had some limitations that should be addressed. The primary limitation of our protocol is inherent to its retrospective design, meaning our analyses were conducted after data collection, which may introduce biases due to potential misalignment with the original study design. In other words, several uncontrolled confounding factors may influence our results, and, as the analysis was not pre-specified, no formal sample size calculation was performed. Moreover, the reasons for MTX discontinuation were not available for all patients due to the nature of this study performed in routine clinical practice. In fact, there was no prospective systematic analysis of specific clinical toxicities of methotrexate at each visit, so some patients may have had toxicities such as oral ulcers not recorded here, and there was no specific recording of the alcohol intake of the patients. Additionally, this study only considered the persistence in MTX therapy, without analyzing the possible effect due to the introduction, during the follow-up, of other drugs such as b/tsDMARDss, steroids, or non-steroidal anti-inflammatory drugs. What concerns steroid use at baseline is that potentially may have a DMARD effect and interfere with MTX survival; however, at Cox regression, no associations were found. Finally, some important clinical data, such as disease activity indices and patient-reported outcomes, were not available for all patients, and, given the nature of this study covering many years, they were assessed using different methods. However, the long-term follow-up in our unit could be a strength of this research. Furthermore, clinical factors such as age, sex, and obesity do not appear to influence the survival rate of MTX in inflammatory arthritis.

## 5. Conclusions

In conclusion, our study confirmed the overall effectiveness and safety of MTX in RA and PsA, with good persistence even in the long term. In fact, at 4 and 8 years from the start of MTX, about 40% and 20% of patients with RA and PsA were still taking MTX, suggesting that despite the broad use of b/ts DMARDs in these diseases, MTX can still represent the “anchor” drug for the treatment of inflammatory joint disease. Future research may evaluate the role of MTX treatment in combination with new b/ts DMARDs in different settings (community, hospital, rehabilitation).

## Figures and Tables

**Table 1:** Clinical characteristics of patients with chronic inflammatory arthritis starting methotrexate (MTX) as first-line treatment.

	RA	PsA	UA
Participants	130	82	30
Age (years), median (IQR)	59 (51–69)	57 (46–65)	53 (46–61)
Male sex, *n*. (%)	25 (24.7)	122 (50.8)	48 (39.3)
Smoking, *n*. (%)	45 (34.6)	24 (29.2)	10 (30)
BMI (kg/m^2^), median (IQR)	25.7 (23.1–29.3)	26.9 (23.8–30.9)	25.1 (21.9–27.5)
Obesity, *n* (%)	13 (12.9)	14 (20.2)	1 (7.1)
SJC, median (IQR)	5 (3–8)	2 (1–4)	2 (1–3)
TJC, median (IQR)	11 (5–17)	4 (2–6)	3 (1–5)
Concomitant treatment at baseline			
- NSAIDs, *n* (%)	22 (16.9)	20 (24.3)	10 (33.3)
- oral steroids, *n* (%)	70 (53.8)	4 (4.8)	9 (30)
- Steroids (methylprednisolone) median dose (IQR) mg	4 (0–4)	4 (4–4)	4 (4–8)
- Duration of steroids treatment; median (IQR), months	6 (3–24)	3 (3–6)	3 (3–6)

Abbreviations: *n*: number; MTX: methotrexate; RA: rheumatoid arthritis; PsA: psoriatic arthritis; UA: undifferentiated arthritis; BMI: body mass index; IQR: interquartile range; NSAIDs: non-steroidal anti-inflammatory drugs; SJC: swollen joint count; TJC: tender joint count.

**Table 2:** Cox regression analysis of predictive factors for MTX survival rate.

Predictive Factors	Hazard Ratio (95% Confidence Interval)	*p*-Value
Age	1.03 (0.78–2.12)	0.42
Sex (male)	1.23 (0.81–2.40)	0.22
Smoking habit	1.52 (0.92–3.34)	0.13
BMI	1.12 (0.86–2.56)	0.65
Concomitant treatment with steroids	0.89 (0.74–1.18)	0.34

BMI: Body mass index.

## Data Availability

The data supporting the findings of this study are available from the corresponding authors upon reasonable request due to privacy/ethical restrictions.
